# Le zona ophtalmique: une dermatose rare chez l'enfant

**DOI:** 10.11604/pamj.2015.22.217.4599

**Published:** 2015-11-10

**Authors:** Douhi Zakia, Marieme Meziane, Gallouj Salim, Mernissi Fatima Zahra

**Affiliations:** 1 Service de Dermatologie-Vénéréologie, CHU Hassan II Fès, Maroc

**Keywords:** Virus varicelle-zona, zona ophtalmique, enfant, Varicella zoster virus, ophthalmic zoster, child

## Abstract

Le zona est dû a une réactivation du virus varicelle-zona (VZV) qui reste quiescent dans les ganglions sensitifs dorsaux après la varicelle. Le zona de l'enfant est rare et particulièrement la forme ophtalmique, qui peut être responsable de complications oculaires graves nécessitant une prise en charge adéquate et précoce. Il est parfois associé à des douleurs post-zostériennes dont le traitement est difficile. L'aciclovir per os administré dans les 72 heures après l’éruption a prouvé son efficacité dans la prévention des complications oculaires. Nous en rapportant un nouveaux cas chez un garçon immunocompétent de 9 ans, sans notion de varicelle antérieure.

## Introduction

Le zona est du a une réactivation du virus varicelle-zona (VZV) qui reste quiescent dans les ganglions sensitifs dorsaux après la varicelle. Le zona de l'enfant est rare et particulièrement la forme ophtalmique, qui peut être responsable de complications oculaires graves nécessitant une prise en charge adéquate et précoce. Nous en rapportant un nouveau cas.

## Patient et observation

**Figure 1:**
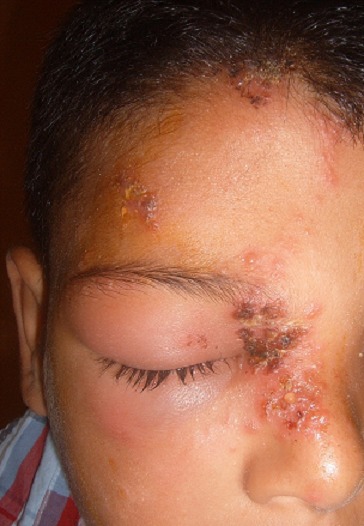
Zona ophtalmique chez un enfant de 9 ans

**Figure 2:**
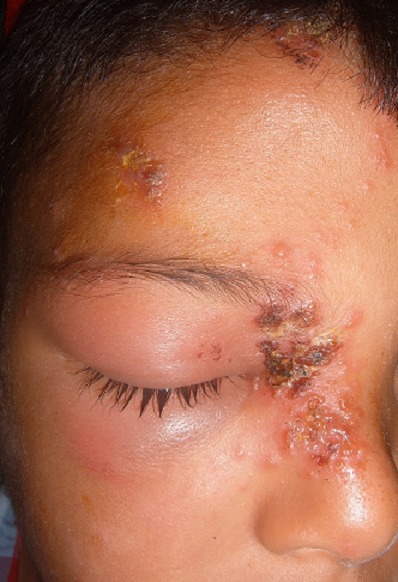
œdème des paupières limitant l'ouverture de l’œil

**Figure 3:**
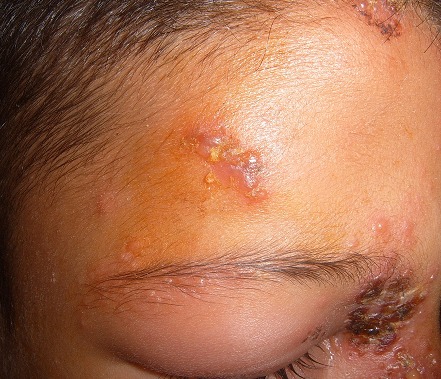
Multiples vésicules groupées en bouquet reposant sur une peau érythémateuse au niveau du versant droit du nez, l'hémi-front droit

Il s'agit d'un garçon de 9 ans, sans antécédent pathologique notable, notamment pas de notion de varicelle néonatale ou maternelle durant la grossesse ou en péri-natal, ni d’épisode similaire; consultait aux urgences pour une éruption douloureuse prenant le front, la paupière supérieure et le nez, évoluant depuis 3 jours. L'examen trouvait un enfant apyrétique avec de multiples vésicules groupées en bouquet, reposant sur une peau érythémateuse, intéressant le versant droit du nez, l'hémi-front droit avec un oedème des paupières supérieure et inferieure droites avec difficulté de l'ouverture de l'oeil (Figure 1, Figure 2, Figure 3). L'examen ophtalmologique à la lampe à fente et au fond d'oeil était sans particularité. Le diagnostic d'un zona ophtalmique a été retenu et l'enfant a été mis sous aciclovir par voie orale. L’évolution était marquée par l'amélioration clinique avec la régression de l'oedème et des douleurs. Un bilan immunitaire minimal a été effectué, notamment une numération formule sanguine, une glycémie et une sérologie VIH, se révélant normal. Le recul actuel est de 2 an sans aucune récurrence ni douleur post zostérienne.

## Discussion

Le zona ophtalmique est une forme clinique rare du zona de l'enfant. Dans une étude indienne sur 195 cas de zona, 22 zona ophtalmique ont était trouvé dont 10% était des enfants [1]. C'est une infection potentiellement grave sur le plan fonctionnel, secondaire à la réactivation latente du VZV situé dans le ganglion trigéminé de Gasser, qui migre le long du nerf ophtalmique, branche V1 du nerf trijumeau. Les trois branches du V (frontale, nasociliaire, lacrymale) peuvent être atteintes de façon simultanée ou isolée [2]. Les facteurs de risque du zona de l'enfant ne sont pas clairement connus, mais il n'est pas lié aux affections malignes comme chez l'adulte [3]. Quelques cas de zona du nourrisson ont été rapportés chez les nourrissons ayant la notion de varicelle maternelle pendant la grossesse [1].

Les complications oculaires surviennent dans 50 à 70% des cas, avec un pronostic souvent réservé [4]. Elles sont représentées essentiellement par une kératite, conjonctivite, uvéite, rétinite, nécrose rétinienne, glaucome et nécrose rétinienne. Les complications neurologiques sont possibles, mais heureusement rares; faite de myélite, méningo-encéphalite, paralysie motrice et oculomotrice, dysfonction vésicale et digestive. La particularité de la forme de l'enfant est la prédominance de signes généraux, l’évolution généralement favorable et les algies post-zostériennes qui restent exceptionnelles [2, 5].

## Conclusion

La particularité de notre observation est la survenue de zona chez un enfant immunocompétent, sans notion de varicelle antérieure et la localisation ophtalmique qui reste une forme rare chez l'enfant.
